# Serum neurofilament light chain levels are associated with white matter integrity in autosomal dominant Alzheimer's disease

**DOI:** 10.1016/j.nbd.2020.104960

**Published:** 2020-08

**Authors:** Stephanie A. Schultz, Jeremy F. Strain, Adedamola Adedokun, Qing Wang, Oliver Preische, Jens Kuhle, Shaney Flores, Sarah Keefe, Aylin Dincer, Beau M. Ances, Sarah B. Berman, Adam M. Brickman, David M. Cash, Jasmeer Chhatwal, Carlos Cruchaga, Michael Ewers, Nick N. Fox, Bernardino Ghetti, Alison Goate, Neill R. Graff-Radford, Jason J. Hassenstab, Russ Hornbeck, Clifford Jack, Keith Johnson, Nelly Joseph-Mathurin, Celeste M. Karch, Robert A. Koeppe, Athene K.W. Lee, Johannes Levin, Colin Masters, Eric McDade, Richard J. Perrin, Christopher C. Rowe, Stephen Salloway, Andrew J. Saykin, Reisa Sperling, Yi Su, Victor L. Villemagne, Jonathan Vöglein, Michael Weiner, Chengjie Xiong, Anne M. Fagan, John C. Morris, Randall J. Bateman, Tammie L.S. Benzinger, Mathias Jucker, Brian A. Gordon, for the Dominantly Inherited Alzheimer Network

**Affiliations:** a Department of Radiology, Department of Neurology, Department of Pathology & Immunology, Department of Psychiatry, Division of Biostatistics, Washington University in St. Louis School of Medicine, Saint Louis, MO, USA; b DZNE-German Center for Neurodegenerative Diseases, D-72076 Tübingen, Germany; c Department of Cellular Neurology, Hertie Institute for Clinical Brain Research, University of Tübingen, D-72076 Tübingen, Germany; d Neurologic Clinic and Policlinic, Departments of Medicine, Biomedicine and Clinical Research, University Hospital Basel, University of Basel, CH-4031 Basel, Switzerland; e Alzheimer Disease Research Center and Pittsburgh Institute for Neurodegenerative Diseases, University of Pittsburgh School of Medicine, 4-West Montefiore University Hospital, 200 Lothrop Street, Pittsburgh, PA, USA; f Department of Neurology, Columbia University Medical Center, New York, NY, USA; g Dementia Research Centre, UCL Queen Square Institute of Neurology, London, UK; h Department of Neurology, Massachusetts General Hospital, Harvard Medical School, Boston, MA, USA; i Institute for Stroke and Dementia Research, Klinikum der Universität München, Ludwig-Maximilians-Universität LMU, Munich, Germany; j Department of Neurodegenerative Disease, Dementia Research Centre, UCL Institute of Neurology, London, UK; k Department of Pathology and Laboratory Medicine, Indiana University School of Medicine, Indianapolis, IN, USA; l Department of Neuroscience, Icahn School of Medicine at Mount Sinai, New York, NY, USA; m Department of Neuroscience, Mayo Clinic, Jacksonville, FL, USA; n Department of Radiology, Mayo Clinic, Rochester, MN, USA; o Department of Radiology, University of Michigan, Ann Arbor, USA; p Department of Psychiatry and Human Behavior, Butler Hospital, Warren Alpert Medical School, Brown University, Providence, RI, USA; q German Center for Neurodegenerative Diseases (DZNE), Munich, Germany; r The Florey Institute, University of Melbourne, Parkville, VIC, Australia; s Department of Molecular Imaging & Therapy, Austin Health, Melbourne, Australia; t Department of Neurology, Butler Hospital, Warren Alpert Medical School, Brown University, Providence, RI, USA; u Department of Neurology, Department of Radiology, Indiana University School of Medicine, Indianapolis, IN, USA; v Banner Alzheimer's Institute, Phoenix, AZ, USA; w Departments of Psychiatry, Radiology, Medicine, and Neurology, University of California at San Francisco, San Francisco, CA, USA; x Department of Neurology, Mayo Clinic, Jacksonville, FL, USA; y Department of Neurology, Klinikum der Universität München, Ludwig-Maximilians-Universität München, Munich, Germany; z Munich Cluster for Systems Neurology (SyNergy), Germany

**Keywords:** Neurofilament, Alzheimer's disease, Neurodegeneration, White matter, Blood-based biomarkers, Neuroimaging

## Abstract

Neurofilament light chain (NfL) is a protein that is selectively expressed in neurons. Increased levels of NfL measured in either cerebrospinal fluid or blood is thought to be a biomarker of neuronal damage in neurodegenerative diseases. However, there have been limited investigations relating NfL to the concurrent measures of white matter (WM) decline that it should reflect. White matter damage is a common feature of Alzheimer's disease. We hypothesized that serum levels of NfL would associate with WM lesion volume and diffusion tensor imaging (DTI) metrics cross-sectionally in 117 autosomal dominant mutation carriers (MC) compared to 84 non-carrier (NC) familial controls as well as in a subset (*N* = 41) of MC with longitudinal NfL and MRI data.

In MC, elevated cross-sectional NfL was positively associated with WM hyperintensity lesion volume, mean diffusivity, radial diffusivity, and axial diffusivity and negatively with fractional anisotropy. Greater change in NfL levels in MC was associated with larger changes in fractional anisotropy, mean diffusivity, and radial diffusivity, all indicative of reduced WM integrity. There were no relationships with NfL in NC. Our results demonstrate that blood-based NfL levels reflect WM integrity and supports the view that blood levels of NfL are predictive of WM damage in the brain. This is a critical result in improving the interpretability of NfL as a marker of brain integrity, and for validating this emerging biomarker for future use in clinical and research settings across multiple neurodegenerative diseases.

## Highlights

• Serum NfL levels reflect white matter integrity in autosomal dominant Alzheimer disease.
• Associations between NfL and white matter imaging are present throughout all brain regions.
• Longitudinal white matter alterations are associated with changes in blood NfL.
• Results improve interpretability of NfL as a marker of brain integrity.

## 1 Introduction

Neurodegenerative disease biomarkers have important roles in defining disease presence and severity, predicting progression, and monitoring disease-modifying therapies. For clinical and research settings such *in vivo* measures include magnetic resonance imaging (MRI), positron emission tomography (PET), cerebrospinal fluid (CSF) assays, and blood-based tests. Blood-based biomarkers (Lewczuk et al., 2018; Zetterberg and Blennow, 2018) have the advantages of minimal invasiveness, subject acceptability, low cost, as well as accessibility in diverse clinical settings, including lesser developed countries.

Neurofilaments are a component of the cytoskeleton in the neuronal axons and are critical for the radial growth and stability of axons (Barry et al., 2012; Rao et al., 2003). Any pathological process that leads to axonal damage or neuronal death should release neurofilament proteins into the surrounding extracellular fluid. Thus, elevations in neurofilament protein biofluid concentrations are not specific to one disease but are a general indicator of axonal damage. Neurofilaments have one of three basic structures with light, medium, or heavy molecular weights. The majority of work in neurodegenerative conditions has focused on the light subunit (NfL).

CSF and blood-based NfL has been evaluated as a potential fluid biomarker in a wide range of neurodegenerative disorders (see Bridel et al., 2019; Gordon, 2020; Khalil et al., 2018) including frontotemporal dementia (FTD) (Meeter et al., 2016; Rohrer et al., 2016; Skillbäck et al., 2014), progressive supranuclear palsy (PSP) (Hansson et al., 2017; Holmberg et al., 1998), amyotrophic lateral sclerosis (ALS) (Gaiottino et al., 2013; Lu et al., 2015), Parkinson's disease (PD)(Hansson et al., 2017), multiple sclerosis (MS) (Chitnis et al., 2018; Disanto et al., 2017; Kuhle et al., 2019), vascular dementia (VAD) (De Marchis et al., 2018; Skillbäck et al., 2014), dementia with Lewy bodies (DLB) as well as sporadic and autosomal dominant Alzheimer's disease (AD) (Gaiottino et al., 2013; Mattsson et al., 2019; Preische et al., 2019; Weston et al., 2017, Weston et al., 2019).

Despite the growing usage of NfL as a biomarker, only a modest number of studies have related NfL levels to the markers of white matter (WM) health to which it should be intimately related and mechanistically linked. Results from MS populations have shown that increased levels of NfL in the CSF or blood were related to greater WM hyperintensity (WMH) volumes and gadolinium enhancing lesions (Chitnis et al., 2018; Dalla Costa et al., 2019; Disanto et al., 2017; Kuhle et al., 2019). A similar pattern was found in patients with ischemic stroke (Tiedt et al., 2018) and cerebral autosomal dominant arteriopathy with subcortical infarcts and leukoencephalopathy (CADASIL) (Gravesteijn et al., 2019). Diffusion tensor imaging (DTI) is a particular form of diffusion-weighted imaging that characterizes the movement of water molecules in the brain and provides a way to examine microstructural changes of WM integrity. Prior studies found that higher levels of NfL are related to worse WM health as captured by higher levels of mean diffusivity (MD) and lower fractional anisotropy (FA) (Menke et al., 2015; Mielke et al., 2019; Moore et al., 2018)although this is not always the case (Mielke et al., 2019; Racine et al., 2019).

Autosomal dominant AD (ADAD) is a form of AD caused by heritable mutations in genes that are involved in the production of beta-amyloid (Aβ). The young age at onset (30–60 years) of ADAD means individuals are largely free of age-related comorbidities (*i.e.* vascular health) that can contribute to WM disease. The relatively predictable age of dementia onset in ADAD also means that one can align asymptomatic individuals relative to their estimated disease onset. This makes it possible to investigate decades' worth of the disease course from large cross-sectional samples. Such studies of ADAD have shown that CSF and blood NfL levels are elevated in symptomatic individuals and begin to increase 10–20 years before symptom onset (Preische et al., 2019; Sánchez-Valle et al., 2018; Weston et al., 2017), consistent with the notion that WM damage is an early event in AD. There is an emerging recognition that frank WM lesions as well as changes observed with DTI are a core feature of ADAD (Araque Caballero et al., 2018; Lee et al., 2016). As a result, ADAD can serve as a model to test whether elevated levels of NfL are related to changes in WM over the course of the disease. In the current work we test the sensitivity of NfL as a measure of white matter decline in neurodegenerative disorders. We hypothesize that serum levels of NfL are associated with WM hyperintensity lesion volume and diffusion metrics in both cross-sectional and longitudinal cohorts with ADAD.

## 2 Materials and methods

### 2.1 Participants

Participants were from the Dominantly Inherited Alzheimer Network (DIAN) observational study recruited from 14 study sites in the USA, UK, Germany, and Australia. DIAN participants are from families with known mutations in presenilin 1 (*PSEN1*), presenilin 2 (*PSEN2*), and amyloid precursor protein (*APP*) genes and have a 50% risk of inheriting the mutation from their affected parent at a relatively similar, and therefore predicable, age at onset within families. Participants who completed genetic, clinical, neuroimaging, and blood draw assessments, and whose data passed quality control as part of the 11th DIAN data release were considered for this study. The sample was restricted to those who had at least one serum NfL measurement, one DTI scan, and one T2-weighted fluid-attenuated inversion recovery (FLAIR) scan within 1 year of serum NfL measurement. The average number of days between blood draw for NfL and DTI scan was 0.92 ± 0.66 (mean ± SE) days. This sample included a subset of DIAN participants previously described (Preische et al., 2019).

The final cross-sectional sample consisted of data from 117 mutation carriers (MC; 87 PSEN1, 12 PSEN2, and 18 APP) and 84 familial non-carrier (NC) controls. Of the 117 MC with baseline data, 41 had two or more visits with serum NfL measurement, DTI scan, and FLAIR scan available for longitudinal analyses (one had four visits, four had three visits, and 36 had two visits).

The institutional review board at Washington University in St. Louis provided supervisory review and human studies approval. Participants or their caregivers provided written informed consent in accordance with their local institutional review board. Participants' relevant background characteristics are listed in Table 1.

**Table 1:** Baseline sample characteristics.

Characteristic	Non-carrier (*N* = 84)	Mutation-carrier (*N* = 117)	*p*-Value
Age (yrs), mean (SD)	40.5 (10.7)	38.6 (10.8)	.230
Sex, female (%)	58.3	50.8	.293
Years of Education, mean (SD)	14.5 (3.2)	14.1 (3.2)	.492
Body Mass Index, mean (SD)	28.8 (6.0)	30.2 (24.2)	.601
aHypertension, %	16.7	6.8	.080
aDiabetes, %	2.4	0.0	.230
Serum NfL (pg/mL), mean (SD)	23.7 (12.5)	33.4 (23.1)	.001
EYO (yrs), mean (SD)	–	-8.5 (11.0)	–
MMSE, mean (SD)	29.2 (1.2)	27.2 (4.1)	<.001
Clinical Dementia Rating, 0, 0.5, ≥ 1, *n* (%)	81 (96.4), 3 (3.6), 0 (0)	76 (65.0), 32 (27.4), 9 (7.6)	<.001

NfL = Neurofilament light chain; EYO = estimated years from expected symptom onset; MMSE = Mini-Mental State Examination.

a Percent of individuals currently receiving active management and/or medication for disease.

### 2.2 Clinical

Cognitive and functional status was assessed using the Clinical Dementia Rating (CDR). ‘Presymptomatic’ was defined as CDR = 0, and ‘symptomatic’ as CDR > 0. For each visit, a participant's estimated years from expected symptom onset (EYO) was calculated based upon the participant's current age relative to either the family mutation-specific expected age at onset of cognitive symptoms or parental age at first progressive cognitive decline if onset for the mutation was unknown. EYO was established identically for both MC and NC family members. The presence or absence of an autosomal dominant AD mutation was determined using PCR-based amplification of the appropriate exon followed by Sanger sequencing (Bateman et al., 2012). Clinical evaluators were blind to the mutation status of participants.

### 2.3 Imaging

DIAN Imaging data was screened for protocol compliance and artifacts. All sites used a 3 T scanner, which was qualified for use at study initiation and was required to pass regular quality control assessments. Volumetric T1-weighted images (repetition time = 2300 ms, echo time = 2.95 ms, flip angle = 9°, 1.1 × 1.1 × 1.2 mm^3^ resolution) were acquired for all participants and were processed using FreeSurfer 5.3 (http://surfer.nmr.mgh.harvard.edu/) (Fischl, 2012) and the Desikan atlas to produce regional estimates of grey matter for use in PET processing.

To characterize diffusion, whole brain DTI data were acquired using T2*-echo planar imaging with one reference volume (b0, b = 1000 mm^2^) and 64 diffusion directions (repetition time = 6000/7800/11000 ms, echo time = 87/85 ms, flip angle = 90°, b-value = 1000/s mm^2^ 2.5 mm isotropic voxels). Only DTI data collected on a Siemens scanner was included in the current study.

### 2.4 DTI preprocessing

Preprocessing included correction for motion and eddy-current distortions followed by skull stripping with FMRIB software library (FSL) 5.0.9. Rigorous motion inspection was applied after eddy-current correction. As described above, participants' data underwent rigorous inspection for artifacts, including determination of motion, which was defined as those who moved >3.5 mm in more than 10% of the diffusion directions. Thirty-five individuals were excluded from the current study due to motion artifacts.

The diffusion tensor model was fit using dtifit within the FMRIB's Diffusion Toolbox included in FSL. Fractional anisotropy (FA) measures how much water movement is restricted to one primary direction. FA ranges from zero to one, with zero being complete isotropic diffusion and one being anisotropic diffusion. Axial diffusivity (DA) and radial diffusivity (DR) respectively reflect the movement of water parallel and perpendicular to (axis of) the fiber bundle. Mean diffusivity (MD) reflects the total amount of diffusion present in all directions. Generally, higher FA and lower MD is thought to represent healthier WM integrity. FA images from all subjects were nonlinearly aligned to the FMRIB58 atlas, which is a diffusion specific template in MNI space, and averaged to create a mean FA image. A skeletonized atlas was generated from this mean image using a threshold of 0.2, which excluded any voxels not overlapping in at least 80% of participants. Each diffusion metric (FA, DA, DR, and MD) was smoothed with a 2-mm kernel and projected onto the skeletonized atlas using the nonlinear registration. Voxel-wise analyses were performed on the skeletonized maps using tract based spatial statistics (TBSS) in FSL (Smith et al., 2006).

### 2.5 DTI regions of interest creation

Well-studied, anatomically derived tracts provide an alternative approach to voxel-wise analyses. For this approach, all DTI metrics were also analyzed using previously defined regions of interest (ROIs) (Strain et al., 2018) from a group of younger adults from a separate cohort (Van Essen et al., 2013). Briefly, deterministic tractography was performed with the MedINRIA software in native space, and each participant's tracts were then transformed to MNI space. Tracts were combined across individuals and limited to only those voxels present in a majority of individuals. This resulted in the creation of 20 tracts including cingulum (left and right), superior and inferior longitudinal fasciculus (left and right), corticospinal (left and right), frontal aslant tract (left and right), perforant pathway (left and right), uncinate fasciculus (left and right), fronto-occipital fasciculus (left and right), forceps major, forceps minor, anterior corpus callosum, and posterior corpus callosum. This atlas includes several WM tracks that are commonly evaluated for DTI analyses and was generated from ROI's described in a prior study (Wakana et al., 2007). Similar white matter tracks are described in more common atlases like the JHU atlas (Oishi et al., 2011).

These WM ROIs were overlaid to the TBSS derived skeletonized atlas and the averaged WM metrics (FA, MD, DA, and DR) were calculated in each ROI. Laterality differences were not expected so the left and right side were averaged together, as applicable, resulting in a total of 12 final ROIs, to decrease the number of comparisons. A relationship between NfL and DTI in this population was expected but the spatial topography was unknown. Therefore, we did not limit our analyses to any particular WM track but instead assessed several WM tracks that covered different areas of the brain to isolate a spatial relationship between DTI and NfL.

MD, DA, and DR values were re-scaled by a factor of 1000 before being entered into our analyses in order to generate more interpretable regression coefficients.

### 2.6 WM hyperintensities

WMH were quantified on FLAIR scans (repetition time = 9000 ms, echo time = 90 ms, TI = 2500 ms, flip angle = 150°, 0.9 × 0.9 × 5.0 mm^3^ resolution) using maps generated with the open-source lesion segmentation tool for SPM that includes a lesion growth algorithm (Schmidt et al., 2012). This algorithm identifies voxels likely to be WMH. For the current analyses, we used the global volume of identified WMH. WMH volumes were not normally distributed, thus a log-transformation was applied.

### 2.7 Aβ-amyloid PET

Aβ-amyloid (Aβ) PET imaging was performed after a bolus injection of [^11^C] Pittsburgh Compound B (PiB). Acquisition consisted of a 70-min scan starting at injection or a 30-min scan beginning 40 min post-injection. Data in the 40–70 min post-injection window were converted to regional standardized uptake value ratios (SUVRs) relative to the cerebellar grey matter using FreeSurfer-derived ROIs, (Su et al., 2013) and were partial volume corrected using a regional spread function technique (Rousset et al., 1998; Su et al., 2015). A global measure of mean cortical uptake of Aβ burden was derived from cortical regions previously shown to have elevated signal in AD (Su et al., 2013). Global Aβ positivity was defined as a mean cortical SUVR ≥1.42 (Mishra et al., 2018; Su et al., 2019; Sutphen et al., 2015; Vlassenko et al., 2016).

### 2.8 Serum NfL measurements

All available DIAN serum samples through the 11th annual data release were shipped to the University of Tübingen for analysis. These processed data were originally published by Preische and colleagues, and a subset was used in the current study. As previously described (Preische et al., 2019), fluids were collected in the morning under fasting conditions. After blood collection, the tubes were left at room temperature for 30 min to allow clotting, and then centrifuged at 2000 ×*g* for 15 min. Serum was placed into a single transfer tube (#60.541, Sarstedt AG&CO.KG, Nümbrecht, Germany) and immediately frozen on dry ice. NfL measurements were performed using a highly sensitive Single Molecule Array (SIMOA) assay using the capture monoclonal antibody (mAB) 47:3 and the biotinylated detector antibody mAB 2:1 (Uman Diagnostics, Umeå, Sweden). The samples were measured in duplicate on a Simoa HD-1 platform (Quanterix) using a 2-step neat assay. All samples were measured blinded. As NfL levels were non-normally distributed, we applied a log-transformation to this measure.

### 2.9 Statistics

#### 2.9.1 Participant characteristics

To compare background characteristics between mutation MC and NC, we performed *t*-tests and chi-square tests, as appropriate.

#### 2.9.2 White matter hyperintensities within MC

As done in prior analyses examining NfL and WM (Chitnis et al., 2018; Dalla Costa et al., 2019; Gravesteijn et al., 2019; Kuhle et al., 2019), we examined WMH volumes. All linear mixed effect (LME) models were constructed and evaluated using the lme4 and lmerTest packages in the R statistical environment. Log transformed WMH volume was entered as the dependent variable; age, sex, and NfL as fixed effects; a separate term for family was included as a random intercept. This family term represents the specific family a participant comes from. NfL was the predictor of interest. Analyses were performed only in MC since, due to their young age, NC are unlikely to have any WMH; whereas WMH have been shown to be a common feature in ADAD (Lee et al., 2016). We wanted to determine if the relationship between WMH and NfL levels were simply driven by WM microstructural changes as measured by DTI. Therefore, we repeated our original analysis after including a global MD metric as a covariate.

#### 2.9.3 Voxel-wise analyses of DTI

To examine whether the associations between baseline WM metrics of interest and NfL levels varied by mutation status at the voxel level, we implemented linear regression models with skeletonized maps of either MD, FA, DA, or DR as the dependent variable and age at visit, sex, NfL, mutation status, and a NfL x mutation status interaction as predictor terms. The initial focus was on the interaction between NfL and mutation status. Statistical modeling was performed with the Randomise toolbox in FSL (Winkler et al., 2014), a nonparametric statistical approach using permutation testing implemented with 5000 permutations. Significant clusters were identified using threshold-free cluster enhancement (TFCE) with a family-wise error corrected significance level of *p* = .05 (Smith and Nichols, 2009).

Prior work indicates that NfL levels are most informative within MC (Preische et al., 2019), suggesting *a priori* that the interaction term will be highly significant. Therefore, as a follow-up analysis, we examined the relationship between baseline DTI metrics and NfL within each mutation status group to better interpret the interaction and to understand the relationship between NfL and voxel-wise measures of WM within MC and NC, separately. We restricted these analyses to voxels that were significant in the interaction analyses described above. We ran separate models predicting MD, FA, DA, or DR, as a function of age, sex, and NfL.

#### 2.9.4 ROI analyses of DTI within MC

An alternate approach to voxel-wise analyses is to use ROIs representing specific WM tracts. To determine if the relationship between WM integrity and NfL is tract specific, we ran a series of LME models in each ROI treating age, sex, and NfL as fixed effects and including a random intercept for family. Dependent variables were the average WM metrics (FA, MD, DA, and DR) from each ROI. As we primarily expected the effects to be in MCs (Preische et al., 2019), we restricted the analyses to this group.

To correct for multiple comparisons, we implemented a Benjamin-Hochberg procedure with a false discovery rate of 5%. Although analyses were performed for each tract, we focused on the posterior corpus callosum, superior longitudinal fasciculus, and corticospinal tracts for visualization. These regions were selected as exemplar ROIs as: 1) posterior corpus callosum is a region with early WM disruption in ADAD and shows the most robust association between NfL and WM metrics in our primary analyses; 2) superior longitudinal fasciculus is associated with default mode network and executive functioning; and 3) corticospinal tract, as a control, is relatively spared in AD until very late stages.

Prior work has shown dramatic changes in Aβ PET, structural MRI, and WMH (Bateman et al., 2012; Benzinger et al., 2013; Gordon et al., 2018; Lee et al., 2016; McDade et al., 2018) in the DIAN cohort as the disease progresses. In a cohort such as DIAN where there are dramatic changes occurring as the disease progresses there is always a concern that statistical relationships can be observed due to the parallel timing of biomarker changes rather than true biological relationships. To rule out if the association between NfL and DTI was simply driven by other, more overt changes in the disease we additionally include global Aβ-amyloid, total WMH load and precuneus cortical thickness measures. These covariates account for the general disease stage of an individual (Aβ-amyloid), overt white matter lesions (WMH), or atrophy that could drive Wallerian degeneration (structural MRI). Of the 117 MC included in primary analyses, 97 had completed a baseline Aβ PET scan. We repeated our original ROI models, with particular focus on the posterior corpus callosum, superior longitudinal fasciculus, and corticospinal tracts, after adding a global Aβ PET, precuneus cortical thickness, and total WMH volume measures as covariates to examine whether they altered the relationship between NfL and DTI metrics. We additionally provide scatter plots of serum NfL, global Aβ PET, precuneus cortical thickness, and total WMH volume to DR in posterior corpus callosum and correlation matrix in the supplemental material to better interpret the relationship amongst these biomarkers.

#### 2.9.5 White matter integrity markers and NfL across the course of the disease

ADAD has a long preclinical phase evolving over decades (Bateman et al., 2012). To evaluate the relationship of NfL and DTI metrics as a function of disease progression, our MC sample was categorized by baseline CDR score into presymptomatic (CDR = 0, *n* = 76) and symptomatic (CDR > 0, *n* = 28) groups. The presymptomatic group was further subdivided by Aβ positivity into early (summary Aβ SUVR <1.42; Aβ-, *n* = 35) and late (summary Aβ SUVR ≥1.22; Aβ+, *n* = 34) groups. These analyses were restricted to the investigation of FA and MD DTI metrics as these are the most common DTI metrics presented in the literature. We predicted FA and MD values in the posterior corpus callosum using LME models that included sex, NfL, group (presymptomatic Aβ-, presymptomatic Aβ+, and symptomatic), and a NfL x group interaction as the fixed effects and family as the random effect. If a significant interaction was present, we compared the relationship between NfL and the corresponding diffusion metric between each group.

#### 2.9.6 Longitudinal relationship between white matter integrity markers and NfL

Longitudinal data were analyzed using LME models as these models can account for covariance introduced by serial measurements within the model and are more ideally suited for dealing with variability in timing or an unbalanced number of data points. The rate of change in NfL (ΔNfL) for each individual was modeled using an LME with fixed effects of time from baseline in years, and a random intercept for family, as well as random slope and intercept terms for each participant. The ΔNfL for each individual was extracted from the model estimates for subsequent analyses. The ΔNfL was then used for a second LME model where the dependent term for each model was a WM measure of interest (MD, FA, DA, and DR DTI measures in posterior corpus callosum ROI and WMH volume) with fixed effect terms for baseline age, sex, time from baseline, extracted ΔNfL, and a time from baseline x ΔNfL interaction. Models contained random slope and intercept terms for participants and random intercepts for family. The primary term of interest was the interaction between the ΔNfL and the time from baseline term. Models were fitted using lme4 in R. For plotting purposes, LMEs were also used to generate individual rates of change for WM integrity markers (ROI MD, FA, DA, and DR measures and WMH volume).

## 3 Results

### 3.1 Participant characteristics

Demographics are presented in Table 1.

### 3.2 Relationship between NfL and WMH in MCs

**Fig. 1:**
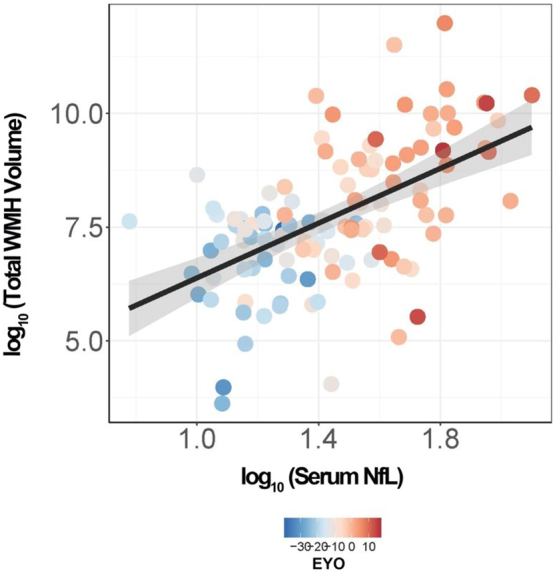
Relationship between serum NfL and total white matter hyperintensity volume in mutation carriers. Scatterplot showing the relationship between total WMH volume and serum NfL in MC (*n* = 117). The shaded area around the linear fit line represents one standard error of the mean from the LME model.

Within the MC cohort there was an association between NfL and total WMH volume (B[SE] = 2.54 [0.56], *p* = 1.44e-05). Fig. 1. When evaluating whether the relationship between NfL and WMH remained after accounting for a global DTI MD measure (as a proxy for overall WM changes measured with DTI) we found the relationship between WMH and NfL was reduced to a trend (B[SE] = 1.15 [0.58], *p* = .05). This suggests that at least a proportion of the association between NfL and WMH is captured by DTI metrics.

## 4 Voxel-wise relationship between NfL and baseline DTI metrics

**Fig. 2:**
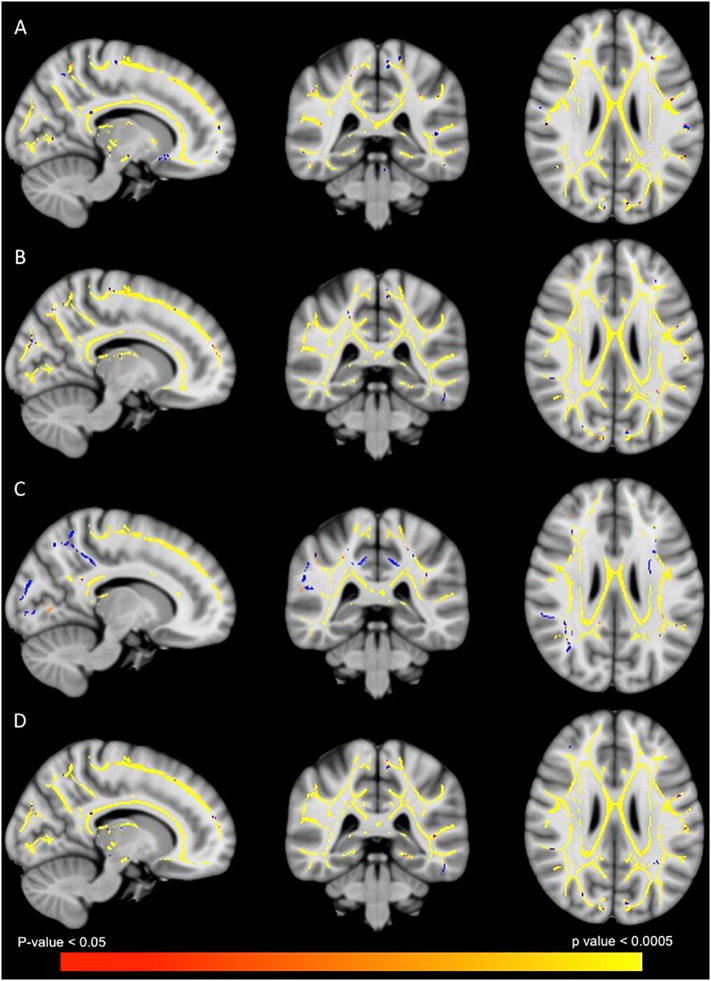
Main Effect of serum NfL on DTI metrics in MC. *p*-Value map (red-yellow) of statistically significant voxel-wise associations of higher NfL and (A) lower fractional anisotropy, (B) higher mean diffusivity, (C) higher axial diffusivity, and (D) higher radial diffusivity superimposed on the white matter skeleton (blue), within mutation carriers (*n* = 117). Familywise error-corrected at *p* = .05. (For interpretation of the references to colour in this figure legend, the reader is referred to the web version of this article.)

There were interactions between NfL and mutation status on all four DTI metrics (FA, MD, axial diffusivity [DA], and radial diffusivity [DR]; see Supplemental Fig. S1). As there was a significant interaction between NfL and mutation status, we next looked within each mutation status group for a main effect of NfL on DTI metrics. Within MCs, a strong association between higher NfL levels and low FA was observed throughout the skeletonized atlas (Fig. 2a). Similarly, there was a strong association between higher NfL levels and higher MD, DA and DR levels across all WM tracts (Fig. 2b–d, respectively). The threshold free cluster enhancement (TFCE) method (see Methods) within our voxel-wise analyses resulted in an inclusive cluster of much of the WM voxels, emphasizing a robust and widespread association between NfL and WM metrics. To better understand subtle tract-specific variations, we subsequently generated a mask containing all significant voxels surviving multiple comparisons correction and applied this mask to our voxel-wise uncorrected statistical map (Supplemental Fig. S2). Within NCs there was no association between NfL levels and FA, MD, DA, or DR across the entire cortex (data not shown).

### 4.1 Regional association between NfL and baseline DTI metrics in MC

**Fig. 3:**
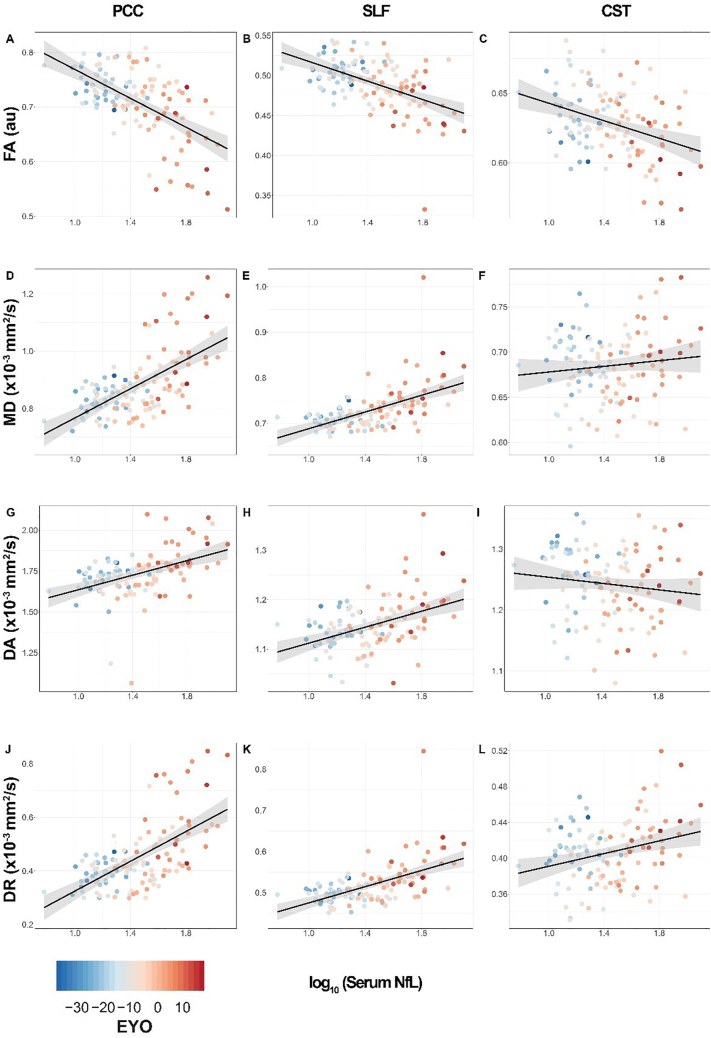
Tract-specific white matter measures are associated with serum NfL in MC. Scatterplots depicting the relationship between serum NfL and DTI metrics from FA, MD, DA, and DR within three representative ROIs (PCC, SLF, and CST) in MC (*n* = 117). The shaded area around each linear fit line represents one SE from LME models. FA = fractional anisotropy; MD = mean diffusivity; DA = axial diffusivity; DR = radial diffusivity; PCC = posterior corpus callosum; SLF = superior longitudinal fasciculus; CST = corticospinal tract; NfL = neurofilament light chain; DTI = diffusion tensor imaging; ROIs = regions of interest; MC = mutation carriers; SE = standard error.

As an alternative to voxel-wise analyses we utilized 12 ROIs that summarized important white matter tracts. There were significant associations between higher NfL levels and lower FA levels across all 12 ROIs examined. There were consistent associations between higher NfL and higher MD, DA, and DR levels in all tracts examined, with the exception of the corticospinal tract. Regression coefficients and *p*-values from statistical models are presented in Table 2. Representative plots are shown depicting the relationship between NfL and FA, MD, DA, and DR in the posterior corpus callosum (Fig. 3a, d, g, and j), superior longitudinal fasciculus (Fig. 3b, e, h, and k), and corticospinal (Fig. 3c, f, i, and l) tracts. For exploratory purposes, to better understand whether there were unique relationships within specific mutation types (*i.e.*, PSEN1, PSEN2, and APP), we evaluated the association between NfL and a singular region and DTI metric of RD in the posterior corpus callosum within each mutation type. (Supplemental Fig. S3).

**Table 2:** Main effect of serum NfL on DTI metrics in MC.

WM tract	Fractional anisotropy		Mean diffusivity		Axial diffusivity		Radial diffusivity	
	B (SE)	*p*	B (SE)	*p*	B (SE)	*p*	B (SE)	*p*
Inferior Longitudinal Fasciculus	−0.062 (0.01)	1.41E-06	0.090 (0.02)	1.61E-05	0.065 (0.02)	.0077	0.102 (0.02)	1.50E-06
Superior Longitudinal Fasciculus	−0.048 (0.01)	.00020	0.091 (0.02)	1.34E-06	0.090 (0.02)	2.71E-05	0.094 (0.02)	4.46E-06
Frontal Occipital Fasciculus	−0.071 (0.01)	1.54E-09	0.108 (0.02)	8.03E-09	0.084 (0.02)	2.32E-05	0.124 (0.02)	6.52E-10
Perforant Pathway	−0.106 (0.02)	5.28E-07	0.150 (0.02)	6.78E-08	0.084 (0.04)	.0193	0.162 (0.03)	6.40E-09
Uncinate Fasciculus	−0.047 (0.01)	4.73E-05	0.060 (0.02)	.00019	0.037 (0.02)	.0398	0.071 (0.02)	3.34E-05
Cingulum	−0.088 (0.02)	7.80E-08	0.133 (0.02)	3.69E-09	0.091 (0.03)	.00123	0.153 (0.02)	1.93E-10
Frontal Aslant	−0.060 (0.01)	9.83E-08	0.089 (0.02)	4.51E-05	0.088 (0.03)	.00197	0.101 (0.02)	6.29E-06
Corticospinal	−0.027 (0.01)	.012	0.032 (0.02)	.0689	0.007 (0.03)	.785	0.042 (0.02)	.00826
Anterior Corpus Callosum	−0.119 (0.02)	3.10E-06	0.284 (0.04)	4.31E-10	0.301 (0.06)	4.52E-07	0.279 (0.04)	7.20E-09
Posterior Corpus Callosum	−0.155 (0.02)	4.26E-10	0.321 (0.04)	1.83E-12	0.276 (0.06)	3.76E-06	0.344 (0.04)	8.09E-12
Forceps Minor	−0.074 (0.01)	1.10E-08	0.099 (0.02)	1.60E-07	0.086 (0.02)	.000364	0.114 (0.02)	1.24E-08
Forceps Major	−0.087 (0.01)	4.77E-09	0.122 (0.02)	2.24E-08	0.082 (0.02)	.000109	0.146 (0.02)	3.14E-09

Unstandardized regression coefficient B and adjusted *p*-values for serum NfL from a series of linear mixed effect models in each ROI, which included random intercepts for family, and fixed effects for age, sex, and NfL. Dependent variables were the average WM metrics (FA, MD, DA, and DR) from each ROI. *N* = 117. WM = White matter; NfL = Neurofilament light chain; ROI = region of interest; SE = standard error.

Including Aβ PET, cortical thickness, and WMH as additional covariates in ROI models for posterior corpus callosum, superior longitudinal fasciculus, and corticospinal tracts in a subset of 97 individuals with Aβ PET available, the relationship between NfL and DTI metrics persisted (*p* values = 5.78e-04, 4.21e-04, 0.020, 4.68e-04 for FA, MD, DA, and DR in posterior corpus callosum, and *p*'s = 0.029, 0.002, 0.038, 0.003 for FA, MD, DA, and DR in superior longitudinal fasciculus). The relationship between FA, MD and DR in corticospinal tract and NfL remained significant (*p*'s = 0.028, 0.046, 0.015, respectively) and relationship between DA in the corticospinal tract and NfL remained nonsignificant in the revised models with Aβ PET and WMH as additional covariates. (Full models in Supplemental Table S6). These results suggest that the association between NfL and FA or MD is not driven solely by Aβ pathology, thinning grey matter, or WMH load.

### 4.2 White matter integrity markers and NfL across the course of the disease

We stratified MC into Symptomatic individuals and Presymptomatic Late (Aβ +), and Early (Aβ-) groups. There was a group x NfL effect on MD (F = 4.3, *p* = .017), DA (F = 4.49, *p* = .014), and a trend for DR (F = 2.52, *p* = .087), but not for FA (F = 0.89, *p* = .415) in the posterior corpus callosum.

Between-group comparisons revealed that the relationship between NfL and MD in the posterior corpus callosum in the Symptomatic group was stronger than that of the Presymptomatic Early group (B [SE] = 0.285 [0.10], *p* = .004), and similar to that of the Presymptomatic Late group (B [SE] = 0.169 [0.10], *p* = .095) (Supplemental Fig. S4). Similarly, the relationship between NfL and DA or DR in the posterior corpus callosum in the Symptomatic group was stronger than that of the Presymptomatic Early group (B [SE] = 0.448 [0.15], *p* = .004 and B [SE] = 0.239 [0.11], *p* = .029, respectively), and not different from that of the Presymptomatic Late group on DA (B [SE] = 0.288 [0.16], *p* = .068) or DR (B [SE] = 0.164 [0.14], *p* = .143) (Supplemental Fig. S4c, d). There were no differences between Presymptomatic Early and Presymptomatic Late groups across any of the DTI metrics examined (*p*'s > 0.278). Along with prior work showing NfL changes in the symptomatic period (Preische et al., 2019), these results suggest that the relationship between NfL and WM is most robust in those with cognitive symptoms but may begin in cognitively normal MCs with significant Aβ pathology and continue or even intensify with the onset of cognitive symptoms.

### 4.3 Longitudinal relationship between white matter integrity markers and NfL

**Fig. 4:**
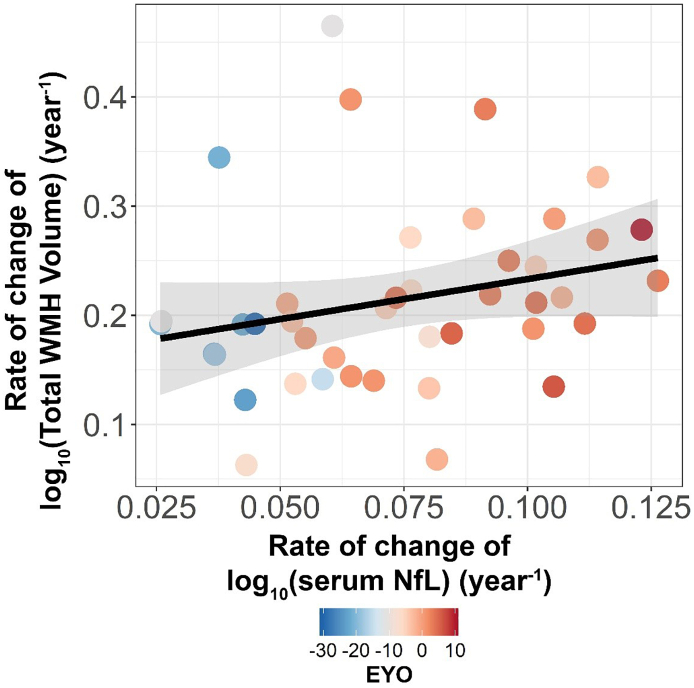
Longitudinal relationship between serum NfL and WMH in MC. Scatterplot showing the relationship between the estimated annual rate of change in total WMH volume and the estimated annual rate of change in NfL in MC (*n* = 41). The shaded area around the linear fit line represents one SE from the LME model. NfL = neurofilament light chain; WMH = white matter hyperintensity; ROIs = regions of interest; MC = mutation carriers; SE = standard error.

**Fig. 5:**
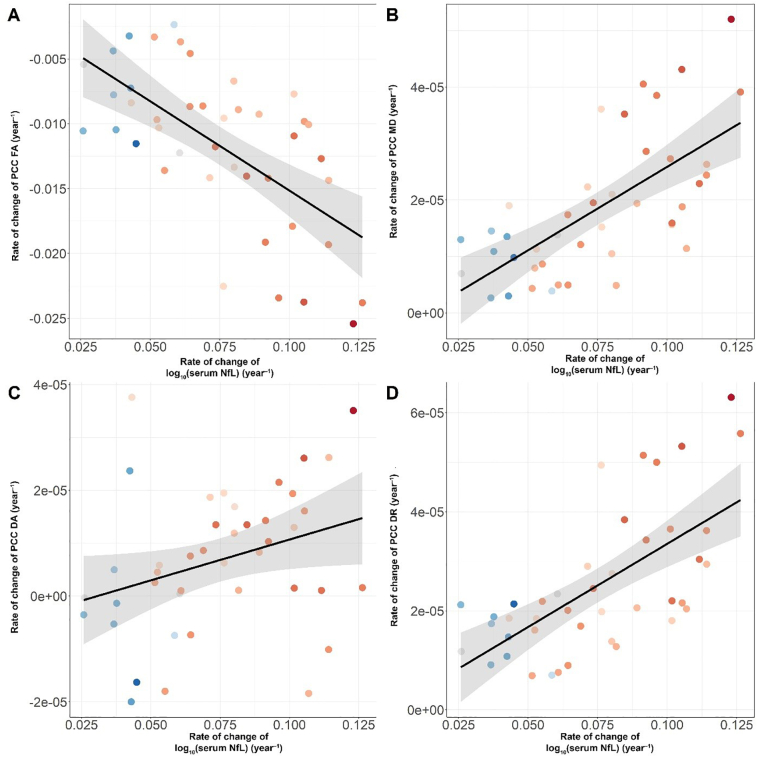
Longitudinal relationship between NfL and DTI metrics in PCC. Scatterplot showing the relationship between the estimated annual rate of change in serum NfL and the estimated annual rate of change in (A) FA in PCC, (B) MD in PCC, (C) DA in PCC, and (D) DR in PCC in MC (*n* = 41). The shaded area around each linear fit line represents one SE from LME models. FA = fractional anisotropy; MD = mean diffusivity; DA = axial diffusivity; DR = radial diffusivity; PCC = posterior corpus callosum; NfL = neurofilament light chain; DTI = diffusion tensor imaging; SE = standard error.

Rate of change in NfL (ΔNfL) was associated with rate of change in FA, MD, and DR in posterior corpus callosum (B[SE] = −0.238 [0.05], *p* = 1.74e-05; B[SE] = 0.0004 [9.0e-05], *p* = 3.02e-04; and B[SE] = 0.0005 [8.64e-05], *p* = 1.42e-06, respectively), but not with rate of change in DA (B[SE] = 0.0002[1.58e-04], *p* = .287) or rate of change in WMH (B[SE] = 1.04[0.65], *p* = .121) (Fig. 4, Fig. 5a–d) consistent with the view that changes in NfL reflect increasing white matter changes in this cohort as measured with DTI.

## 5 Discussion

NfL is a promising fluid biomarker to study neurodegeneration across multiple neurological diseases. Although NfL is thought to reflect damage to large myelinated axons there is a paucity of work systematically examining how NfL levels relate to established markers of macrostructural and microstructural WM damage. In the current work we examined whether levels of serum NfL are a reflection of WM lesion volumes and DTI metrics of WM integrity. Across all measures in MC, we found that elevated levels of serum NfL were significantly associated with increased levels of WM pathology. This indicates that a blood-based measure of NfL does indeed track WM damage in the brain.

Due to its predictable age of dementia onset and low comorbidities, ADAD serves as a useful model to understand the evolution of AD pathology (Bateman et al., 2012; Moulder et al., 2013). Prior work established increases in WMH volumes (Lee et al., 2016) as well as alterations in DTI metrics (Araque Caballero et al., 2018) as core features of ADAD. Across multiple neurological conditions, increased NfL levels in CSF and blood have been tied to greater WMH volumes (Chitnis et al., 2018; Dalla Costa et al., 2019; Gravesteijn et al., 2019; Kuhle et al., 2013, Kuhle et al., 2019). Consistent with this work, we found that in MC, higher serum NfL levels at baseline are associated with greater WMH lesion volumes. However, when we included a global MD DTI measure for microstructural WM integrity as a covariate, the relationship between NfL and WMH was no longer present. This suggests that at least some of the information contained by global WMH volumes is also reflected in DTI metrics. For exploratory purposes, a correlation matrix depicting the relationship between biomarkers examined is presented in supplementary material. In the subset with longitudinal data, there was a trend that a greater increase in serum NfL was related to increases in WMH volume, but this did not approach significance (B[SE] = 1.04[0.65], *p* = .121).

WMH represent macrostructural WM insults in the brain. In addition to such overt damage, microstructural changes in WM can be assayed using DTI. There have been inconsistent results establishing whether CSF and blood levels of NfL are sensitive to such microstructural changes in WM detectable using DTI (Menke et al., 2015; Mielke et al., 2019; Moore et al., 2018; Racine et al., 2019; Tiedt et al., 2018). When examining baseline DTI data using voxel-wise- and ROI-based approaches in MC we found that higher levels of serum NfL were negatively associated with FA, and positively associated with MD, RD, and DA throughout the entire cortex, although effects were strongest in posterior regions (Table 2 and Supplemental Fig. 2). Lower FA and higher MD, RD, and DA are indicative of a less constrained flow of water molecules and are generally viewed as markers of WM damage. This widespread pattern of WM decline is consistent with prior work in ADAD (Araque Caballero et al., 2018). The strong observed relationship suggests that NfL is a robust marker of active microstructural WM damage beyond overt lesions.

As the mutations in ADAD lead to such a dramatic disease phenotype, there is always a concern that significant relationships between biomarkers may be due simply to a common time course rather than measures being truly interrelated. Even when including Aβ PET and cortical thickness as a markers of general disease progression and WMH volume to account for macrostructural WM damage, we still found highly significant associations between serum NfL levels and DTI metrics. The relationship between NfL and DTI was present not only at baseline but was consistent over time. In the subsample of MC with longitudinal data, a greater rate of serum NfL change was associated with greater WM declines in FA, and increases in MD, and RD. Establishing such a longitudinal relationship is critical to be able to use CSF and blood-based measures of NfL to monitor disease progression and to potentially use NfL as a marker of response to disease intervention in clinical trials for AD as well as other neurodegenerative disorders.

The heritability in the onset of dementia in ADAD families provides the unique ability to stage individuals relative to their expected time of cognitive decline. Stratifying by CDR and Aβ positivity status, we were able to examine the relationship between NfL and WM integrity across the course of the disease. In asymptomatic MC without advanced disease progression (Aβ-), levels of NfL were low and WM, assessed with DTI, was healthy. As the disease progressed (Aβ+), levels of NfL increased and WM health declined in individuals who were still classified as asymptomatic but Aβ+. Finally, there were even further increases in NfL and declines in DTI metrics in symptomatic individuals. The distribution of the groups overlapped and within the entire group of mutation carriers NfL and DTI measures were tightly coupled. This suggests that continuous levels of the biomarkers add to the assessment of the health of the brain over and above Aβ positivity status and staging by CDR alone.

The current work is a critical step towards establishing NfL as a marker of neurodegeneration that reflects likely WM damage and decline. Still, there are limitations to the current analyses. ADAD is a continually progressive neurodegenerative condition with relatively stereotyped phenotypes and rates of progression, and manifests during a younger age range, during which, secondary comorbidities are uncommon. These features make ADAD a very useful model of AD pathobiology and a model to test the relationship between NfL and WM damage. However, these features may affect the generalizability of the current findings to late onset AD. For example the degree of AD pathology seen in ADAD is higher than that seen in late onset AD (Gordon et al., 2019), which may make it easier to detect associations. Stroke and ischemia have been shown to increase NfL levels (Moseby-Knappe et al., 2018; Pujol-Calderón et al., 2019), but it is unclear how chronic comorbidities such as obesity, hypertension, and diabetes influence the protein. While such age-related comorbidities are low in ADAD, in sporadic AD they may represent stronger influences on both white matter and NfL levels than primary AD proteinopathies. Given the previous mixed findings examining Nfl and white matter in older adult cohorts (Mielke et al., 2019; Moore et al., 2018; Racine et al., 2019), there may be a lower utility of NfL in sporadic AD.

Although longitudinal relationships between NfL and WM are rarely examined, our longitudinal sample is modest. CSF and blood-based biomarkers reflect properties of the brain and body at the time of collection. Prior work suggested that CSF biomarkers of neuronal injury decline at symptomatic stages of AD (Llibre-Guerra et al., 2019; Sutphen et al., 2018), which could lead to a mismatch between biofluid and imaging markers in later stages of disease. Further work is needed to test whether the relationship between imaging and NfL measures changes at more advanced stages of neurodegenerative conditions.

Finally, further examinations relating neuroimaging and biofluid measures across multiple neurological disorders are needed. In ADAD we showed that neuroimaging and biofluid measure are highly related. Although elevated in most neurodegenerative conditions (Bridel et al., 2019; Gordon, 2020), the degree that NfL becomes abnormal varies widely between disorders. In other diseases the strength of the relationship between neuroimaging and biofluid measures may be more or less robust.

Biomarkers provide the ability to measure the health of the central nervous system *in vivo* to aid disease diagnosis and prognosis. Blood-based markers are minimally invasive and relatively low cost but few have been validated against neuroimaging biomarkers (Lewczuk et al., 2018). NfL in the blood is highly correlated with measures in the CSF and is becoming widely adopted in diagnosing and monitoring multiple diseases. Our current work supports the view that blood levels of NfL reflect WM damage in the brain at least as measured with neuroimaging. This is a critical result in improving the interpretability of NfL as a marker of brain integrity, and for validating this novel biomarker for future use in clinical and research settings.

## Declaration of competing interest

A.M.G. has consulted for Cognition Therapeutics, Biogen, GSK, Illumina, Eisai, AbbVie and Pfizer and served on the SAB for Denali Therapeutics.

## Appendix A Supplementary data

Supplementary material Image 1
