# Supplementary material for: Serum neurofilament light chain levels are associated with white matter integrity in autosomal dominant Alzheimer's disease
Source: Neurobiol Dis. 2020 Aug;142:104960. doi: 10.1016/j.nbd.2020.104960 (PMC7363568; doi:10.1016/j.nbd.2020.104960)
Supplement: Supplementary file 1 — Supplementary material [file mmc1.docx]

**
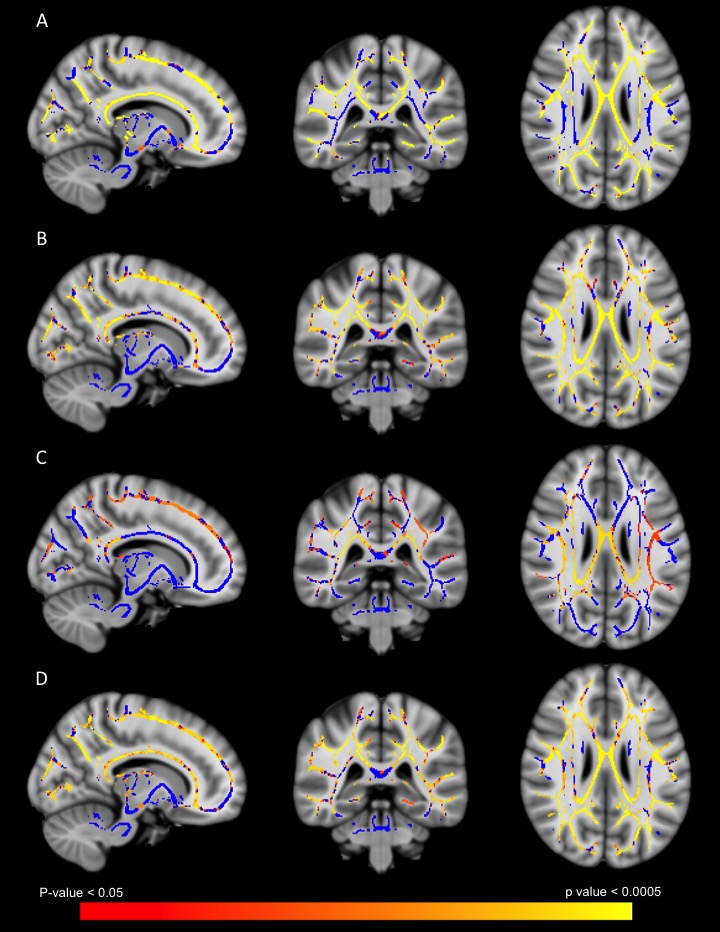
**

**Supplemental Figure S1. Interaction between serum NfL and mutation status on DTI metrics.** P-value map (red-yellow) of statistically significant voxel-wise interactions of NfL x mutation status and (A) lower fractional anisotropy, (B) higher mean diffusivity, (C) higher axial diffusivity, and (D) higher radial diffusivity superimposed on the white matter skeleton (blue). N=201. Family-wise error-corrected at p = 0.05.


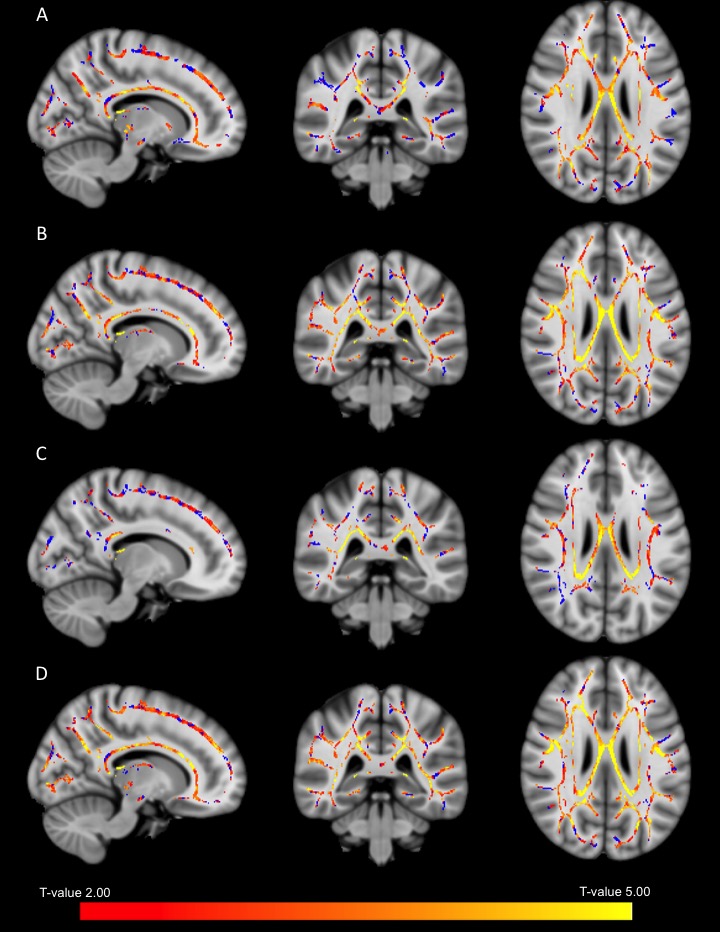


**Supplemental Figure S2. Uncorrected voxel-wise associations of NfL and DTI metrics.**

Uncorrected T-value map (red-yellow) of voxel-wise associations of higher NfL and (A) lower fractional anisotropy, (B) higher mean diffusivity, (C) higher axial diffusivity, and (D) higher radial diffusivity superimposed on the white matter skeleton (blue), within mutation carriers (n=117). Uncorrected maps were restricted to significant clusters identified using threshold-free cluster enhancement (TFCE) with a family-wise error corrected significance level of p = 0.05, presented in Figure 1.

**
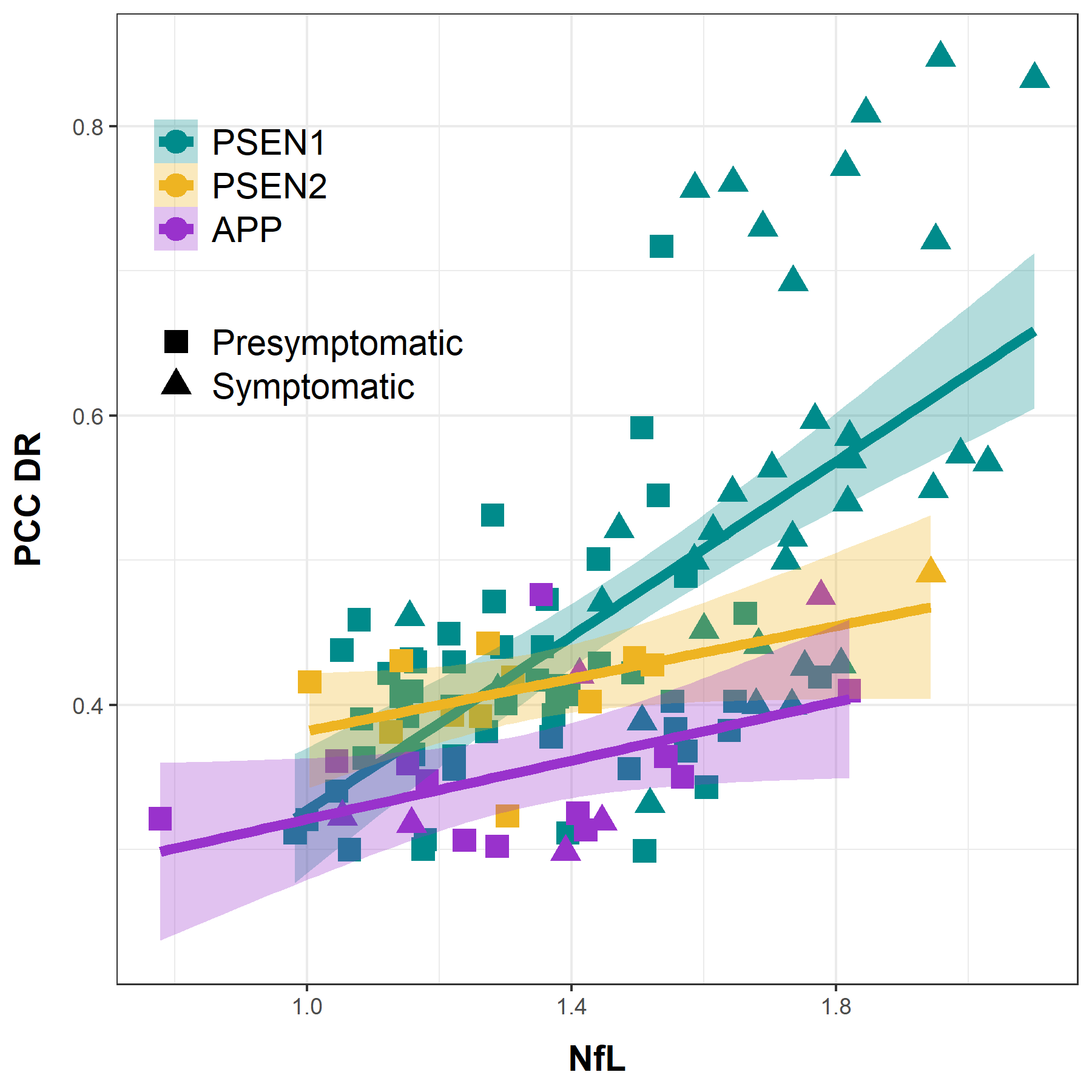
**

**Supplemental Figure S3. Relationship between serum NfL and radial diffusivity within posterior corpus callosum by mutation type.** Graphical depiction, for exploratory purposes, of the relationship between serum NfL and radial diffusivity in the posterior corpus callosum within each mutation type. Those with mutations in PSEN1 (N= 87) are blue, PSEN2 are yellow (N=12), and APP are purple (N=18). Individuals who are Presymptomatic (CDR=0) are coded as squares and Symptomatic (CDR>0) as triangles. The shaded area around each linear fit line represents one SE. DR = radial diffusivity; PCC = posterior corpus callosum; NfL = neurofilament light chain; SE = standard error.

**
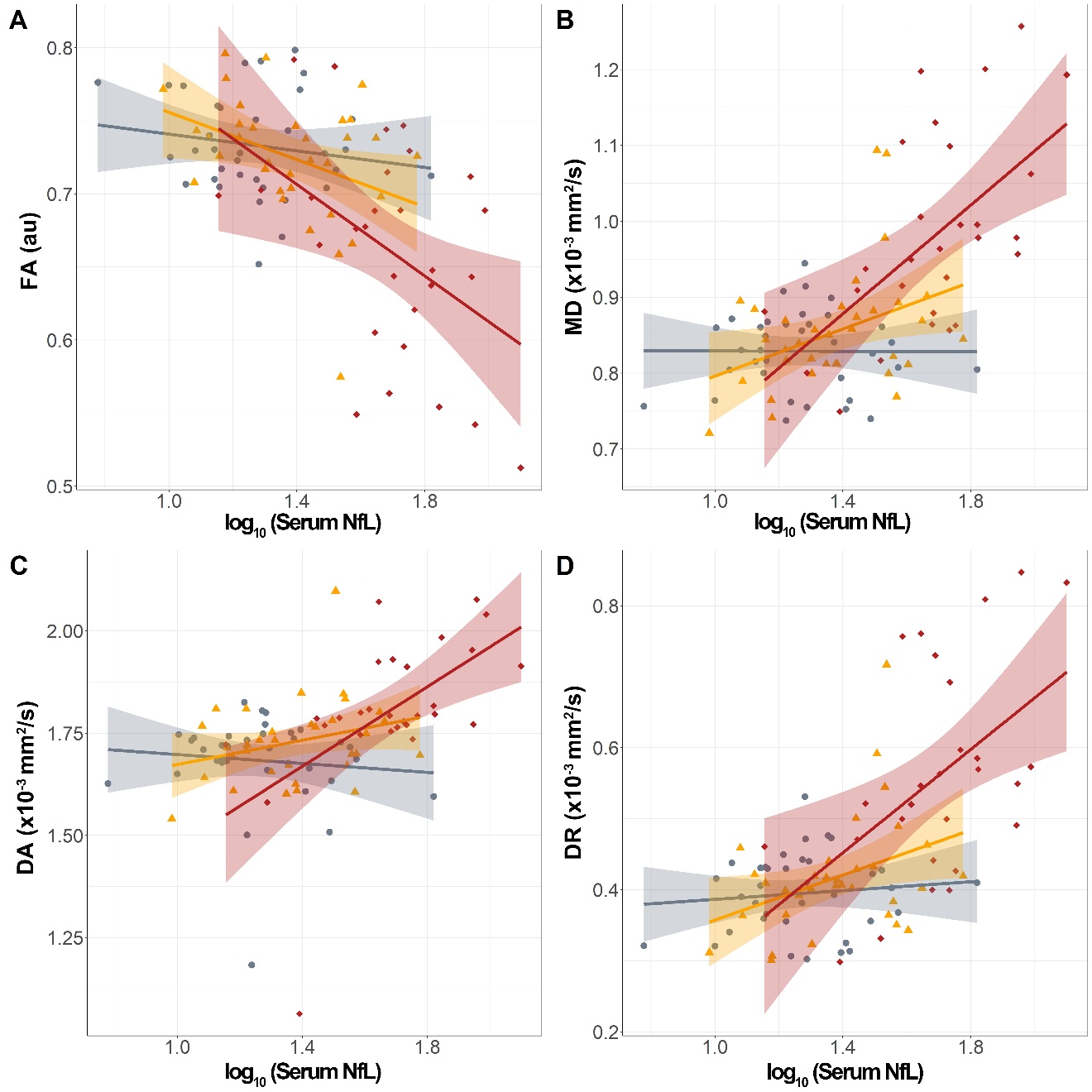
**

**Supplemental Figure S4. Interaction between serum NfL and disease progression on DTI metrics.** Scatterplots depicting the relationship between serum NfL and DTI metrics from FA, MD, DA, and DR within PCC for Presymptomatic MC early (gray circles), Presymptomatic MC late (yellow triangles), and Symptomatic MC (red diamonds).

The shaded area around each linear fit line represents one SE from LME models.

FA = fractional anisotropy; MD = mean diffusivity; DA = axial diffusivity; DR = radial diffusivity; PCC = posterior corpus callosum; NfL = neurofilament light chain; DTI = diffusion tensor imaging; SE = standard error.


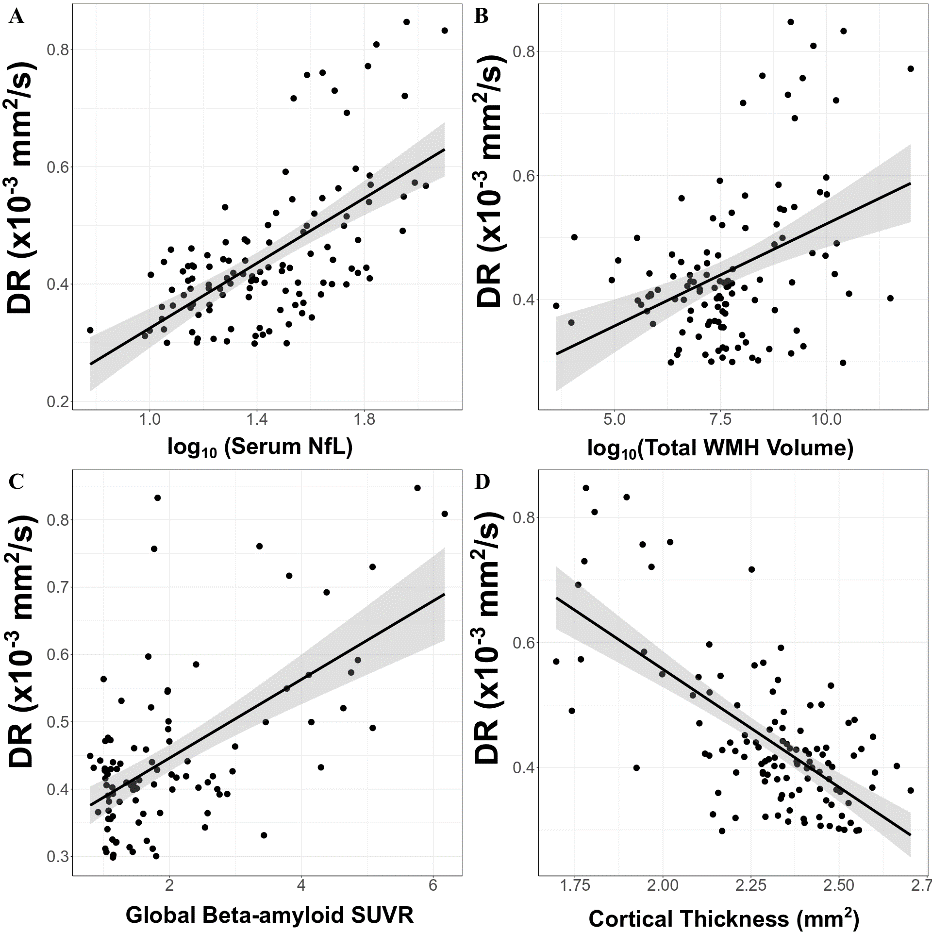


**E**

**
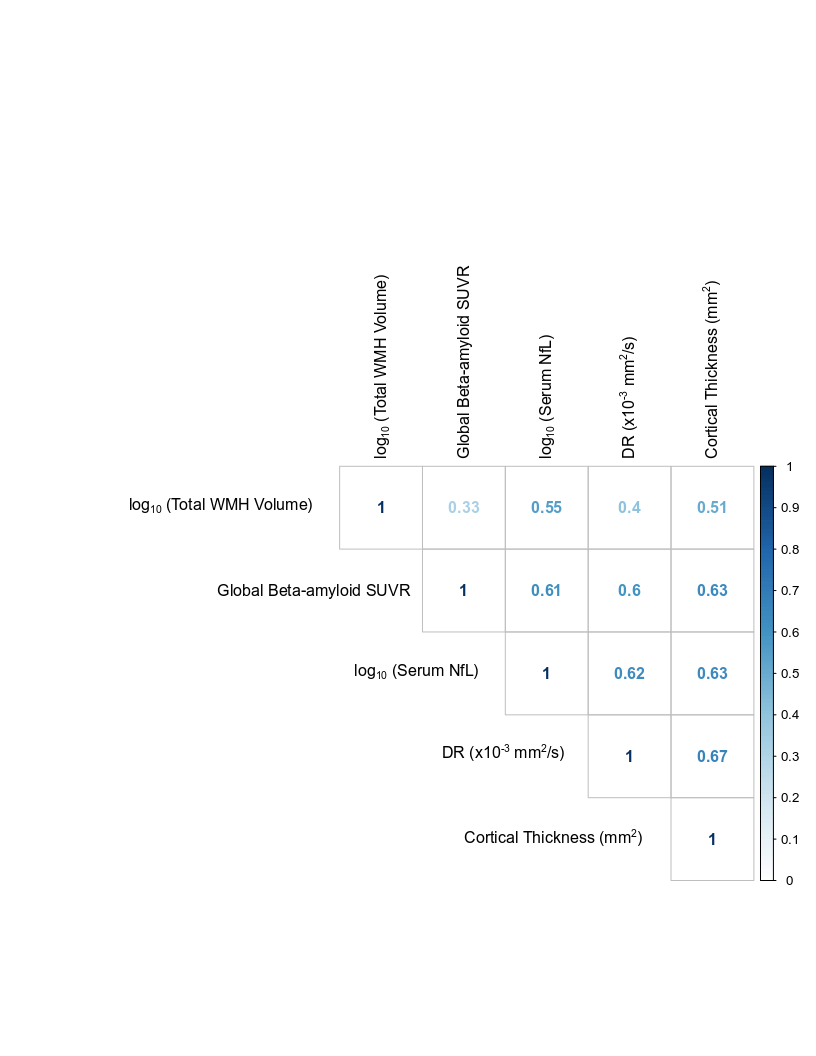
**

**Supplemental Figure S5.** **Relationship between biomarkers and radial diffusivity within posterior corpus callosum in MC.** Depiction of the relationship between radial diffusivity within posterior corpus callosum in MC and serum NfL (A), total WMH volume (B), global beta-amyloid (C), and cortical thickness in the precuneus (D). A correlation matrix (E) of Pearson correlation coefficients amongst all biomarkers considered is presented to better understand these relationships. DR = radial diffusivity; PCC = posterior corpus callosum; NfL = neurofilament light chain; WMH= white matter hyperintensity.

| **Fixed Effect** | **FA in PCC** | | **MD in PCC** | | **DA in PCC** | | **DR in PCC** | |
| --- | --- | --- | --- | --- | --- | --- | --- | --- |
|  | **B (SE)** | **p** | **B (SE)** | **p** | **B (SE)** | **p** | **B (SE)** | **p** |
| **Intercept** | 0.521 (.09) | 5.21e-08 | 1.095 (.15) | 7.69e-11 | 1.700 (.07) | 3.76e-10 | 0.777 (.17) | 1.07e-05 |
| **Sex** | -0.012 (.01) | 0.034 | 0.043 (.02) | 0.006 | 0.016 (.03) | 0.532 | 0.045 (.02) | 0.011 |
| **Age** | 0.001 (.001) | 0.026 | -0.003 (.001) | 0.009 | -0.003 (.002) | 0.062 | -0.003 (.001) | 0.012 |
| **Cortical thickness** | 0.108 (.03) | 2.18e-04 | -0.169 (.05) | 5.72e-04 | -0.054 (.08) | 0.482 | -0.218 (.05) | 8.83e-05 |
| **Aβ-amyloid** | -0.003 (.005) | 0.487 | 0.028 (.01) | 9.96e-04 | 0.048 (.01) | 4.18e-04 | 0.020 (.01) | 0.031 |
| **WMH volume** | 0.004 (.003) | 0.438 | -0.005 (.006) | 0.433 | -0.010 (.01) | 0.305 | 0.020 (.01) | 0.468 |
| **NfL** | -0.097 (.03) | 5.78e-04 | 0.168 (.05) | 4.21e-04 | 0.178 (.07) | 0.020 | 0.188 (.05) | 4.68e-04 |
|  | **FA in SLF** | | **MD in SLF** | | **DA in SLF** | | **DR in SLF** | |
|  | **B (SE)** | **p** | **B (SE)** | **p** | **B (SE)** | **p** | **B (SE)** | **p** |
| **Intercept** | 0.516 (.05) | <2.0e-16 | 0.608 (.06) | <2.0e-16 | 1.029 (.08) | <2.0e-16 | 0414 (.07) | 1.24e-08 |
| **Sex** | 0.002 (.01) | 0.734 | 0.007 (.01) | 0.257 | 0.015 (.01) | 0.071 | 0.003 (.01) | 0.671 |
| **Age** | 5.785e-05 (3.397e-04) | 0.865 | -0.0004 (.0004) | 0.323 | -0.0005 (.0005) | 0.354 | -0.0004 (.0004) | 0.360 |
| **Cortical thickness** | 0.011 (.02) | 0.471 | -0.006 (.02) | 0.762 | -0.0005 (.03) | 0.986 | -0.011 (.02) | 0.590 |
| **Aβ-amyloid** | -1.272e-04 (.003) | 0.963 | 0.002 (.003) | 0.591 | 0.004 (.004) | 0.406 | 0.002 (.004) | 0.595 |
| **WMH volume** | -0.003 (.002) | 0.211 | 0.007 (.002) | 0.004 | 0.006 (.003) | 0.077 | 0.006 (.003) | 0.036 |
| **NfL** | -0.033 (.01) | 0.029 | 0.058 (.02) | 0.002 | 0.054 (0.03) | 0.038 | 0.062 (.020) | 0.003 |
|  | **FA in CST** | | **MD in CST** | | **DA in CST** | | **DR in CST** | |
|  | **B (SE)** | **p** | **B (SE)** | **p** | **B (SE)** | **p** | **B (SE)** | **p** |
| **Intercept** | 0.674 (.05) | <2.0e-16 | 0.645 (.08) | 5.64e-13 | 1.325 (.12) | <2.0e-16 | 0.340 (.07) | 2.11e-06 |
| **Sex** | -0.007 (.005) | 0.142 | 0.026 (.01) | 0.001 | 0.039 (.01) | 0.002 | 0.020 (.01) | 0.005 |
| **Age** | -0.0001 (.0003) | 0.623 | -0.0005 (.0005) | 0.339 | -0.0007 (.0007) | 0.320 | -0.0002 (.0004) | 0.587 |
| **Cortical thickness** | -0.010 (.01) | 0.493 | 0.015 (.02) | 0.537 | 0.020 (.04) | 0.598 | 0.014 (.02) | 0.517 |
| **Aβ-amyloid** | -0.001 (.002) | 0.638 | 0.002 (.004) | 0.644 | -0.0004 (.006) | 0.949 | 0.003 (.004) | 0.457 |
| **WMH volume** | 0.003 (.002) | 0.196 | -0.008 (.003) | 0.014 | -0.011 (.005) | 0.035 | -0.006 (.003) | 0.034 |
| **NfL** | -0.031 (.01) | 0.028 | 0.047 (.023) | 0.046 | 0.030 (.036) | 0.404 | 0.051 (.02) | 0.015 |

**Supplemental Table S6.** Unstandardized regression coefficient and adjusted p-values for fixed effects from a series of linear mixed effect models in PCC, SLF, and CST, which included random intercepts for family, and fixed effects for age, sex, precuneus cortical thickness, global Aβ-amyloid, total WMH volume, and serum NfL. Dependent variables were the average WM metrics (FA, AD, DA, and DR) from each ROI. N=97.

WMH = White matter hyperintensity; NfL = Neurofilament light chain; FA = fractional anisotropy; MD = mean diffusivity; DA = axial diffusivity; DR = radial diffusivity; PCC = posterior corpus callosum; SLF = superior longitudinal fasciculus; CST = corticospinal tract.
